# A Six-Step Protocol for Monitoring Antimicrobial Resistance Trends Using WHONET and R: Real-World Application and R Code Integration

**DOI:** 10.3390/mps8050115

**Published:** 2025-10-02

**Authors:** Fabio Ingravalle, Antonio Vinci, Marco Ciotti, Carla Fontana, Francesca Pica, Emanuele Sebastiani, Clara Donnoli, Martino Guido Rizzo, Dario Tedesco, Silvia D’Arezzo, Stefania Cicalini, Michele Tancredi Loiudice, Massimo Maurici

**Affiliations:** 1 Department of Biomedicine and Prevention, Tor Vergata University of Rome, 00133 Rome, Italyantonio.vinci@students.uniroma2.eu (A.V.); sebastiani@agenas.it (E.S.); 2 Virology Unit, Polyclinic Tor Vergata Foundation, 00133 Rome, Italy; marco.ciotti@ptvonline.it; 3 Microbiology and Biobank Unit, National Institute for Infectious Diseases “Lazzaro Spallanzani”, IRCCS, 00149 Rome, Italy; carla.fontana@inmi.it (C.F.); silvia.darezzo@inmi.it (S.D.); 4 Department of Experimental Medicine, Tor Vergata University of Rome, 00133 Rome, Italy; pica@uniroma2.it; 5 Research and International Relations Unit, Italian National Agency for Regional Healthcare Services, 00187 Rome, Italy; donnoli@agenas.it (C.D.); loiudice@agenas.it (M.T.L.); 6 Department of Public Health and Infectious Diseases, Sapienza University of Rome, 00185 Rome, Italy; martino.guidorizzo@uniroma1.it; 7 Department of Innovation in Healthcare and Social Services, Emilia Romagna Region, 40127 Bologna, Italy; dario.tedesco@regione.emilia-romagna.it; 8 Department of Medicine and Surgery, University of Parma, 43125 Parma, Italy; 9 Systemic and Immune Depression-Associated Infections Unit, National Institute for Infectious Diseases “Lazzaro Spallanzani”, IRCCS, 00149 Rome, Italy; stefania.cicalini@inmi.it

**Keywords:** antimicrobial resistance, surveillance, R programming, microbiology laboratory data, open-source tools

## Abstract

Antimicrobial resistance is a global health issue, and the WHO has made significant efforts in the development of tools for its monitoring. However, such tools are underutilized, due to limited knowledge, technical capacity, and scarcity of economic resources. AMR surveillance can be conducted using WHOnet and R, two free-of-charge software tools widely adopted in both clinical practice and scientific research. WHOnet is designed for managing laboratory data and antimicrobial susceptibility test results, while R is a programming language dedicated to statistical computing and data visualization. The combined use of these tools enables a reproducible workflow for retrospective AMR trend analysis. This paper provides step-by-step instructions on how to perform such analysis and also provides the respective R code. The described code and software results are shown using real-world data from an Italian hospital as an example. The standardization of the analysis process and the rapid availability of data on antimicrobial resistance are critical for both clinicians and public health professionals. They would allow for empirical decisions on antimicrobial treatment based on the specific epidemiological characteristics of the hospital or community setting.

## 1. Introduction

### 1.1. Antimicrobial Resistance as a Global Health Issue

Antimicrobial resistance (AMR) poses a significant challenge to global Public Health. The prevalence of antimicrobial-resistant microorganisms is increasing even in developed countries, while in low- and middle-income nations, AMR represents a great burden due to limited resources and healthcare infrastructures [1,2].

Over the past few decades, the constant increase in AMR rates has led the WHO to declare that “Antimicrobial resistance threatens the effective prevention and treatment of an ever-increasing range of infections caused by bacteria, parasites, viruses and fungi”. This is not only a concern for human health, but also for environmental and animal health, because the use of antimicrobials is widespread in multiple activities beyond the healthcare field [3].

According to estimates from the OECD, in high-income healthcare settings the incremental cost attributable to an infection caused by multidrug-resistant bacteria ranges between approximately €8500 and €34,000 per episode, with considerable variability across countries and clinical contexts [4]. In 2015, it was observed that 671,689 infections and 33,110 deaths from antibiotic-resistant bacteria occurred in Europe [5].

The increasing prevalence of AMR is a complex and multifactorial phenomenon that requires a one health approach. Furthermore, many definitions describing the degree of antibiotic resistance—such as Multi-Drug Resistant (MDR), Extensively Drug Resistant (XDR), Pan Drug Resistant (PDR), and Difficult-to-Treat Resistant (DTR) are assigned to microorganisms that are pathogenic to humans, but not necessarily to those affecting animals or the environment. Among the variety of clinical microbiology methods used for antibiotic susceptibility testing, minimum inhibitory concentration (MIC) assays have become the gold standard in clinical practice [3,6,7].

AMR diffusion is an issue in both hospital, territorial, and non-clinical settings. In hospitals, AMR causes prolongation of stay and increases complication rates and expenditure; even in the Western world, this puts a strain on health systems that, especially in the public sector, are often overburdened, and underfunded [8,9].

Outside hospital settings, AMR also represents an alarming concern, with resistance to commonly used antibiotics being reported even in remote areas and already widespread in primary care. This phenomenon is largely driven by the high and often inappropriate consumption of antimicrobials in healthcare, including community and primary care prescribing, as well as by non-healthcare activities such as agriculture, veterinary medicine, and other human uses that contribute to the global rise and geographic convergence in antibiotic [10,11,12,13]. The effective management of AMR in hospitals, as well as in the community, is challenging and requires a global strategy; therefore, the UN General Assembly formalized a Global Action Plan to tackle AMR in 2016 [14].

Current surveillance systems are slow and struggle to keep pace with the dynamic changes in health care. Although case-based surveillance approaches and systems using real-time geotemporal analysis have been developed to support real-time surveillance, technical and regulatory challenges, as well as difficulties in promoting meaningful cooperation among different stakeholders, have hindered One Health approaches towards AMR control [15].

Several national and international projects aim to conduct comprehensive health surveillance; among them. The European Antimicrobial Resistance Surveillance Network (EARS-NET) and European Surveillance of Antimicrobial Consumption Network (ESAC-NET) curate population level data over time and throughout the European Economic Area [16]. Similar networks have been established in Asia, such as the Japan Nosocomial Infections Surveillance (JANIS), and in the Americas, such as the Brazilian ResistNet program [17,18].

However, the tools developed for such projects are not widespread in local facilities and are not frequently used on a local level. The barriers towards complex AMR surveillance systems are mostly due to the cost, the usage of proprietary software, the lack of a definite standard in laboratory machinery output format data, and the ultimate fragmentation of reporting tools. Operationalization of integrated surveillance for AMR is still not well established on a global scale, especially in low- and middle-income countries [19,20].

### 1.2. The WHOnet Software

WHOnet v.25.04.25 is a free Windows-based database software, developed for the management of microbiology laboratory data and the analysis of antimicrobial susceptibility test results. It is available from the World Health Organization website in 45 different languages, with modules for laboratory configuration, data entry, data analysis, public health reporting, and data encryption, among others [21]. In several countries, it has been used for years to detect outbreaks using antimicrobial resistance phenotypes [22,23,24,25].

Objectives of WHOnet include

- Enhancing the local use of laboratory data for guiding therapy, assisting infection control, characterizing resistance epidemiology and identifying laboratory testing errors.
- Promoting collaboration in surveillance activities through the exchange of data.

WHOnet can be used for manual data entry, especially in laboratories without an existing computer system for microbiology data. For laboratories which do have systems for managing their data, the BacLink v.25.04.25 software is used for extraction and conversion of data from many different sources (commercial database and spreadsheet software, commercial susceptibility test instruments, and hospital and laboratory information systems) into WHOnet. Usually, BacLink downloads and installs automatically along with the WHOnet software; it is also available free of charge from WHO website [26].

### 1.3. R Software

R version 4.4.0 is a programming language for statistical computing and data visualization. It is widely used in the scientific field due to its great versatility and the possibility of customizing the results [27,28]. R has a native command line interface, but third-party graphical user interfaces are available, such as Rstudio [29].

### 1.4. Purpose of This Paper

This paper provides a step-by-step indication of how to perform an analysis of antimicrobial trend in any setting, from a small-scale laboratory to a nation-wide network, visualizing a simple regression output by pathogen-microbial coupling. An example of end-results using real-world data, retrospectively collected for epidemiological purposes, is also provided, as well as the Excel and R code used for analysis (Appendix A and Appendix B). Providing an end-to-end guide (from raw lab outputs to publication-ready reports and figures) will lower the technical entry barrier for low-resource facilities and public health Authorities, especially settings with scarcity of economic and/or personnel resources.

## 2. Experimental Design

This paper describes the usage of WHOnet and R in a retrospective design, focusing on AMR resistance rates over time. The analysis and data visualization can be used in both observational and non-observational studies, and data can be classified depending on setting, exam type, hospital/ward specialty, or any other user-provided definition.

In our example, we classified data according to period (quarterly). Data can of course be aggregated in many ways, and monthly, weekly, or even daily analysis can be performed, if sufficient data are available.

The regression-based analysis shown at the end can be used by researchers as a quick exploration, and to evaluate long-term trend in AMR behavior. Further investigation can be carried out using the same processed data and more sophisticated techniques, if needed. These techniques include time-series analysis, whose description is beyond the scope of this paper.

For WHOnet to work correctly, it is necessary to use the BacLink software to transform the laboratory-native file format (for example, .csv or .txt or a specific format of the laboratory software) into a file format accepted by WHOnet.

In this paper, we describe the use of WHOnet in native settings to ensure a rapid analysis of antimicrobial resistance found in the samples during the observation period.

A test sample of 105 *S. aureus* isolates, together with their corresponding antibiograms, was systematically collected between 1 January and 31 December 2020. The isolates were grouped by quarter for the calculation of antibiotic resistance rates. For the purposes of this protocol, levofloxacin was selected as the reference antibiotic for analysis. Breakpoint interpretation was performed according to EUCAST guidelines. These resistances, observed over time, were subsequently imported into R to perform an analysis of the observed trends. R software was also used to facilitate the graphical representation of the trends in antimicrobial resistance.

### Materials and Equipment

The WHOnet and BacLink softwares are designed to run natively on the Windows Operating System. For Mac, Linux, Unix, or other OS users, there are still some options to use WHOnet, but they all rely on running or accessing a Windows interface from a non-Windows computer [30].

The software requirements to reproduce the steps described in this paper are minimal, and are commonly available and of widespread usage:

- **Any data management software** (for this paper, we used Microsoft Excel^®^ V.2504, but any other software with similar functionalities can be used effectively);
- **WHOnet software** (WHOnet V.25.04.25, freeware, available from WHO) [31];
- **BacLink software** (BacLink V.25.04.25, freeware, is usually downloaded and installed automatically with WHOnet; it can also be downloaded from WHO) [31];
- **R software** (R version 4.4.0 and R-Studio 2025.05.0 Build 496, freeware, available from the R Foundation and Posit^®^ website) [32].

## 3. Procedure

In this section, we describe the mandatory steps needed to reproduce an analysis which results in information on resistance trend across time, for each pathogen-antimicrobial coupling of choice. Both WHONET and R allow extensive customization and versatility; for such optional analyses, researchers should refer to the technical documentation provided with the respective software. These analyses may also include stratification by patient- or context-specific variables—such as gender, age, race, or geographic and climatic factors—although these aspects were beyond the scope of the present protocol.

The steps for the outbreak monitoring analysis proposed in this paper are as follows:

1. Data extraction from microbiology laboratory software;
2. Data import with BacLink;
3. Configuration and import of data in WHOnet;
4. Data analysis in WHOnet;
5. Preparation for analysis in R;
6. Data Analysis in R.

**Figure 1:**
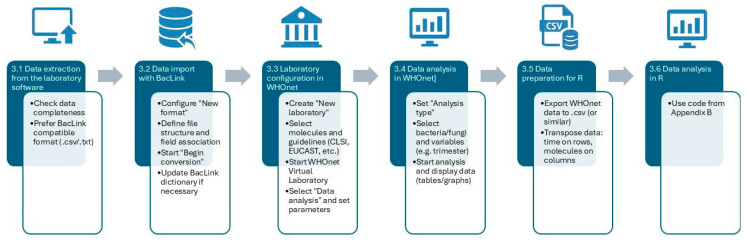
The six steps required to correctly perform the analysis.

These steps are sequential, and the flow is summarized in Figure 1.

### 3.1. Data Extraction from Microbiology Laboratory Software


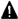
 **CRITICAL STEP**. Data extraction from laboratory software is a variable procedure, as it depends on the platform adopted in each laboratory. In our context, it was available Vitek 2 (bioMérieux, Inc., Hazelwood, MO, USA) and Matrix-Assisted Laser Desorption/Ionization—Time of Flight (MALDI-TOF, Bruker Daltonics, Bremen, Germany). This phase is critical because an incorrect or incomplete export may lead to data loss or compromise subsequent steps of the protocol. The investigator responsible for the export must always verify that all variables of interest—such as isolate identification number, sampling date, microorganism name, and corresponding antibiotic susceptibility results—are present and correctly formatted.

When possible, the preferred option is to export the dataset in a format natively supported by BacLink. If such a format is unavailable, alternative formats such as .csv or .txt may be used (In this protocol we deal with this eventuality). These formats have the advantage of being easily viewable and editable with most data management software. Regardless of the format, it is strongly recommended to perform a visual inspection of the exported file before importing it into BacLink, checking for completeness of records, consistency of delimiters, and correct alignment of rows and columns.

### 3.2. Data Import with BacLink


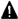
 **CRITICAL STEP**. **When importing data for the first time**, it is necessary to create a new format using the BacLink Configuration wizard. This tool enables the configuration of laboratory data for import into WHONET. Through the “File structure” option, the user specifies the file type, the file path, and the antimicrobial information, and links the relevant variables from the source file to the corresponding “Data fields” of the new file to be imported into WHONET. The “New data file” option allows the selection of the path and format for the newly generated file.

Once the wizard has been configured, the user can confirm the name and format of the new data file and initiate the “Begin conversion” process. During this step, BacLink displays the first three rows of the database both before and after the transformation. If no further action is required, the software proceeds to transform the entire file. Any terms not recognized by the native BacLink dictionary—such as non-standard names of antimicrobials, microorganisms, or other variables—are flagged at the end of the first conversion. At this point, the user may update the BacLink dictionary, ensuring that subsequent transformations are automated and eliminating the need to repeat the configuration. This procedure facilitates faster and more consistent imports in future analyses.

In this protocol, a researcher manually verified the integrity of the data in the imported .csv file and the exported .dbf file, both before and after the BacLink conversion. Performing such a verification at least once, even in other contexts, should be considered good practice to minimize the risk of errors.

### 3.3. Configuration and Import of Data in WHOnet


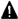
 **CRITICAL STEP**. Before importing the file generated with BacLink into WHONET, the software must first be configured to receive data from the converted .dbf file. WHONET provides the option to configure a virtual laboratory through the “New laboratory” function. This configuration tool enables researchers to define the molecules of interest (e.g., antibiotics or antifungals) and to select the interpretive guidelines to be applied, such as CLSI, EUCAST, or other standards. The selection of “Antibiotics” is mandatory, as it defines the antimicrobial panel for analysis. Other options, such as “Location”, “Data fields”, and “Alerts”, are optional and allow customization or stratification of the dataset if these variables have been previously set.

Once all the molecules have been included (in this protocol, the antibiotics tested in the antibiogram), the user must ensure that the virtual laboratory configuration has been correctly saved and is accessible within WHONET. This check is particularly important when multiple virtual laboratories have been created. To verify or load a virtual laboratory, the user navigates to the “File” menu and selects “Open laboratory”.

After confirming that the virtual laboratory is correctly loaded, the corresponding data can be accessed through the “**Data entry**” menu. This menu becomes available only if the virtual laboratory has been successfully opened. By selecting the “**Open data file**” option, the user can choose the file previously prepared in Step 2 (BacLink conversion) and saved on the local computer. In this protocol, the file used had the **.dbf** extension, retrieved from the designated save path.

### 3.4. Data Analysis in WHOnet


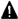
 **CRITICAL STEP**. Once the configuration described in the previous step has been completed, data analysis can be performed by selecting the “Data analysis” menu. This menu allows the user to choose among the different types of analyses available in WHONET (for a comprehensive list, please refer to the official WHONET technical documentation). Within this menu, the “Analysis type” option enables the evaluation of resistance for each bacterium or fungus against the previously configured molecules.

In this protocol, we performed a “%RIS and test measurements” analysis. This option allows the assessment of resistance rates for the antibiotics tested (default setting) and their distribution over time, stratified by quarters (defined in the “Rows” section).

WHONET also offers the possibility of stratifying the dataset according to additional variables, such as the clinical department, specimen type, or other user-defined parameters, if available in the imported dataset.

After completing the configuration, the analysis can be executed. The results are displayed within WHONET but can also be copied or exported in both database and graphical formats. The output displays observed cases stratified by organism, molecule, and time period (quarters in our example). Data may be presented either as absolute numbers or as percentages of the total number of cases that meet the criteria defined in the selected rows (in our case, Staphylococcus aureus, the specific antibiotic, and the corresponding quarter).

For molecules with defined breakpoints according to the guidelines selected during laboratory configuration (Step 3), WHONET provides resistance rates, 95% confidence intervals, and the distribution of results around the breakpoint, available in both tabular and graphical formats.

### 3.5. Preparation of Data for Analysis in R


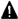
 **CRITICAL STEP**. To import the data into R, the WHONET output must be saved in .csv or another compatible database format. In this protocol, a worksheet was structured with time intervals arranged in rows and the antimicrobial agents investigated listed in columns. The resistance values retrieved for each molecule within the corresponding time interval were entered into the table cells.

The final output is exemplified in Table 1. The transposition formula applied in Microsoft Excel, together with a detailed explanation and an illustration of the format used, is provided in Appendix A.

### 3.6. Data Analysis in R

To analyze the data, the script provided in Appendix B must be executed in the R console. This code is designed to perform a time-dependent linear regression for all variables listed in the columns prepared as described in Appendix A. For each variable, the script generates a scatter plot with the regression line and its 95% confidence interval. In addition, each plot displays the corresponding regression equation, along with the R^2^ value and the *p*-value of the regression model.

## 4. Expected Results

### 4.1. WHOnet Outputs

**Figure 2:**
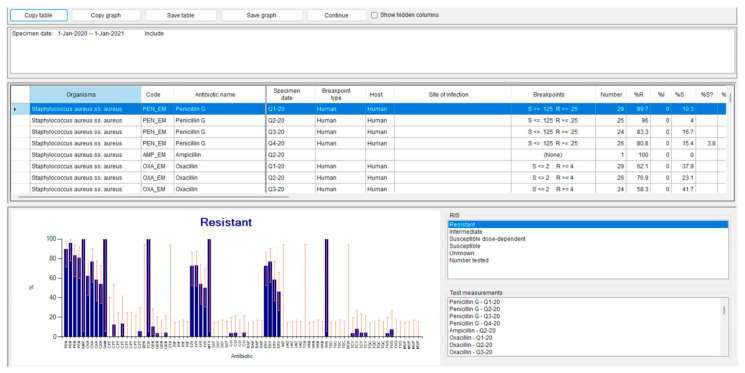
WHOnet output with resistance analysis by organism type, molecule and quarter analyzed. The blue columns represent the percentage of samples resistant to a given antibiotic, while the orange lines indicate the error bars.

When the described steps are completed, the analysis performed with WHONET allows the visualization of the distribution of **antimicrobial** resistance for each analyzed pathogen, as illustrated in Figure 2. These figures display the results generated by WHONET during the analysis described in **Step 3.4**. Through the “**RIS**” menu, it is possible to examine the overall levels of resistance and susceptibility for each antimicrobial agent.

**Figure 3:**
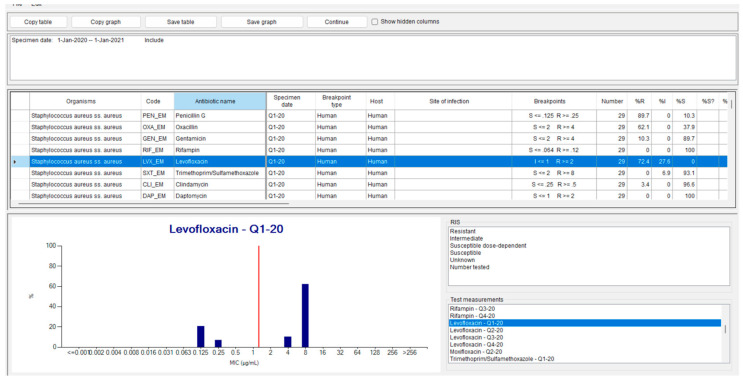
WHOnet output with the distribution of MICs detected in the first trimester for *S. aureus* versus levofloxacin. The blue columns represent the percentage of samples resistant to a given antibiotic, while the orange lines indicate the error bars.

Through the WHONET navigation menus, users can visualize resistance distributions and obtain detailed information on the breakpoint-based classification for each antimicrobial agent. The “**Test measurements**” menu provides access to the distribution of MIC values by molecule and by quarter. Figure 3 illustrates this functionality, showing how the resistance of the *Staphylococcus aureus* sample is distributed with respect to the breakpoints for levofloxacin, as interpreted according to the **EUCAST 2025 guidelines**.

### 4.2. R Outputs

The R analysis provides the results of the linear regression directly in the R console, including the regression coefficient, its 95% confidence interval, and the corresponding R-squared and *p*-value. In this protocol, we employed a simple linear regression model; however, the script provided in Appendix B can be further customized to accommodate more complex models if required. This type of analysis allows us to identify whether resistance rates are increasing over time (positive and significant beta coefficient), whether they are decreasing (negative and significant beta coefficient) or whether the trend is uncertain (non-significant beta coefficient).

**Figure 4:**
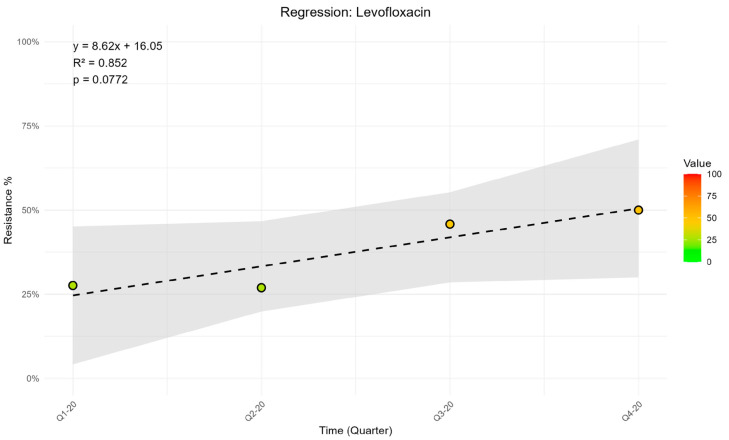
Time-dependent linear regression of Levofloxacin resistance observed in *S. aureus* during the investigation period.

In addition, the script generates a graphical output in the form of a scatter plot, displaying the distribution of the resistance rates for each antibiotic under study. For illustrative purposes, the example presented in this protocol refers to Staphylococcus aureus tested against levofloxacin (Figure 4; R^2^ = 0.852, *p* = 0.0772). The figure consists of a scatter plot with observed resistance percentages on the y-axis and time (quarters) on the *x*-axis. The corresponding regression line is superimposed, together with the 95% confidence interval band and the regression parameters (slope, intercept, R^2^, and *p*-value).

## 5. Discussion

### 5.1. Strengths and Limitations

Although the trend analysis presented in our protocol is for illustrative purposes only, it demonstrates the feasibility of implementing an AMR surveillance scheme based on empirical data derived from microbiology laboratory software.

This approach has the potential to deliver several important benefits for both clinicians and public health professionals. Surveillance of antimicrobial resistance is often limited by the availability of resources and the complexity of analytical tools; however, the protocol we propose can be implemented even in local or resource-limited contexts. This is possible because the workflow is built entirely on open-source software: both WHONET v.25.04.25 and R version 4.4.0 are freely available, do not require licensing fees, and can run on standard computer hardware, thereby reducing economic and technical barriers.

The innovative aspect of our work is not simply the use of WHONET and R together—something that has already been explored in other studies—but the creation of a reproducible, end-to-end pipeline that integrates the strengths of both tools into a single, structured workflow. WHONET is particularly advantageous because it is intuitive and designed specifically for microbiological data management, making it accessible to laboratory staff for routine analyses. R, on the other hand, provides advanced statistical methods and high-quality graphical outputs, which are essential for interpreting resistance trends in a robust and visually clear way. By combining the two, our pipeline ensures that both everyday laboratory needs and more complex analytical requirements are met.

To make this integration practical and reproducible, we included two additional components: a standardized Excel-based transposition step (Appendix A), which ensures that the data exported from WHONET can be consistently reformatted for further analysis, and a customizable R script (Appendix B), which automates the regression analyses and the generation of graphs. These elements were introduced because, in our experience, data exported from different laboratory information systems may vary in structure, and without a standardized step users may struggle to reproduce results across settings.

Another strength of our protocol is its feasibility in low-resource environments. Because both WHONET and R are free and widely distributed, they do not require financial investment. Furthermore, their computational demands are minimal, meaning that the protocol can be executed on ordinary computers without the need for advanced infrastructure. This lowers the entry barrier for institutions that might not otherwise have access to sophisticated analytical platforms (on-site or cloud).

Alternative workflows, such as the direct use of R on raw CSV or Excel exports, often require advanced programming and data management skills that may not be available in all laboratories. In contrast, the proposed protocol was designed to demand only basic computer skills, making it accessible to non-specialists while still ensuring robust and reproducible results.

The proposed pipeline presents several favorable aspects. Owing to its modularity and flexibility, the method allows modification of the data aggregation strategy (e.g., analyses by month, by Gram classification, or by other user-defined criteria) as well as customization of the selected data (e.g., patients from specific settings such as home or nursing facilities, infections occurring more than 48 h after hospital admission, or cases restricted to particular wards or divisions). The approach is also broadly generalizable, since input data can originate from any microbiology laboratory. For this reason, it is essential that operators carefully define the selection of input cases and apply the appropriate breakpoints according to the guidelines adopted in their specific setting.

For all these reasons, we believe that the described protocol offers an effective balance between accessibility, reproducibility, and analytical power, making it a useful tool for supporting AMR surveillance in a wide variety of settings. Furthermore, the command scripts that have been developed for analysis are publicly available for peer-verification (Appendix A and Appendix B). Moreover, for organizations that have sufficient technical capacity to implement one-click reporting, R-code customization or macro implementation in WHOnet is an added value.

Some possible limitations to the implementation of this protocol should also be mentioned. The main limitation is the need to check the input data and for any missing or incorrect data in the microbiology laboratory application. The results in case of faulty data could be inconsistent or unreliable. However, for this eventuality WHOnet provides the confidence intervals of the resistances to the molecules and the code provided in Appendix B automatically excludes a specific analysis when the data are missing, as in the case of non-sampling for a given molecule or the absence of a certain microorganism. Secondly, for clinical implementation, knowledge in clinical microbiology/infectious disease is required, since it is necessary to interpret the analysis of both in vitro and in silico results to apply it to the specific patient’s situation (for example, not all molecules can be used for that type of infection or based on patient’s clinical conditions). Another limitation of the application of this protocol could be determined by the lack of personnel trained in the use of R and R-studio, although the script in Appendix B minimizes the researcher actions necessary to replicate the results of this protocol. Nonetheless, AMR routine monitoring could be used on a local level to implement policies and procedures linked to locally observed AMR epidemiology, on a facility or even ward basis, for example, when providing suggestions for empirical antibiotic therapy.

It is important to recognize the limitations of applying linear regression to time-dependent AMR data. The assumptions required for valid inference, such as homoscedasticity, normality of residuals, and an adequate sample size, may not always be satisfied. In our protocol, the variation over time was not intended to predict future resistance rates but rather to retrospectively examine their fluctuations. These results should therefore be considered illustrative and always interpreted in the context of additional factors that influence AMR dynamics, including antimicrobial use, infection control practices, and the potential impact of clonal spread. Users are strongly encouraged to verify that the underlying assumptions are met before applying this method to their own datasets.

### 5.2. Enhancing Infection Management Through Data-Driven Protocols and Telemedicine

The application of this protocol, or similar protocols, data-mining, rapid analysis and data monitoring could have a strong impact on clinical and public health practice. Local analysis could show in each setting the differences with what is reported by the public health agencies and would allow clinicians to implement empirical treatment lines based on the local circulation of microorganisms and their resistance [33].

**Figure 5:**
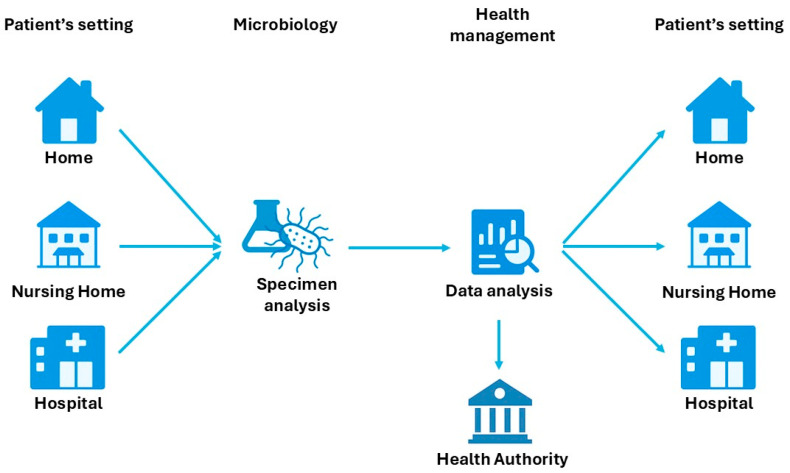
Hypothetical representation of the flow related to microorganism samples. The data analyzed by the laboratory can be re-elaborated centrally and made available to both health authorities and clinicians in their respective care settings.

In a possible context of raw data originating from peripheral structures (for example home, nursing home and small community hospitals) and centralized analysis (especially in the case of a centralized microbiology laboratory, which serves these peripheral structures), it would also be possible to implement the construction of a digital remote communication network to support infection control and antibiotic stewardship (Figure 5).

Moreover, a coordinated infection surveillance into both communities and hospital wards, with tools originally developed to enhance patient monitoring and clinical decision-making, has opened new possibilities for applying digital health infrastructures to support infection control and antimicrobial stewardship efforts. Different studies have demonstrated that continuous monitoring of patients can be extended beyond hospital walls. This approach can also strengthen infection control measures, allowing early recognition of potentially transmissible conditions and bridging gaps between community-based care and hospital-level interventions [34,35].

## 6. Conclusions

The use of the described protocol would allow monitoring the evolution of AMR even in settings with low availability of resources. The sharing of information about resistance and its evolution is fundamental to guide the clinician in the choice of personalized therapy, even of an empirical type, while waiting for the results of the targeted therapy. The centralized management of this information by the public health authority would enable clinicians to receive guidance based on the same data originating from patients in those settings. Free software solutions and the ability to monitor AMR dynamics, almost in real time, should be key in implementing this protocol.

## Figures and Tables

**Table 1:** Table of data ready for R analysis.

Time Period Label	Time ID ^1^	Antibiotic 1	Antibiotic 2	Antibiotic …
Period 1	1	data	data	…
Period 2	2	data	data	…
…	…	…	…	…

^1^ Time ID must be in consecutive order.

**Figure A1:**
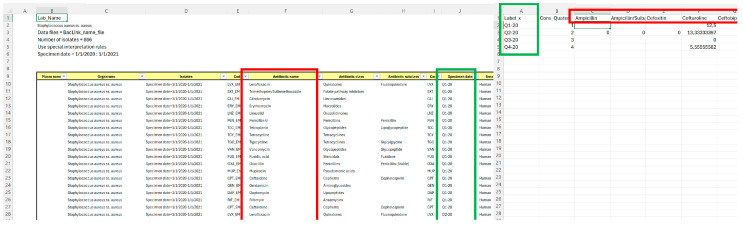
On the left the sheet exported from WHOnet, on the right the construction of the transposed matrix.

## Data Availability

The original contributions presented in this study are included in the article. Further inquiries can be directed to the corresponding author.

## Abbreviations

The following abbreviations are used in this manuscript (alphabetical order):

AMR	Antimicrobial resistance
CLSI	Clinical and Laboratory Standards Institute
DTR	Difficult-to-Treat Resistant
EARS-NET	European Antimicrobial Resistance Surveillance Network
ESAC-NET	European Surveillance of Antimicrobial Consumption Network
EUCAST	European Committee on Antimicrobial Susceptibility Testing
JANIS	Japan Nosocomial Infections Surveillance
MDR	Multi-Drug Resistant
MIC	Minimum Inhibitory Concentration
PDR	Pan Drug Resistant
UN	United Nations
WHO	World Health Organization
XDR	Extensively Drug Resistant
%RIS	Percentage of Resistant, Intermediate and Susceptible

## Appendix A

Figure A1 shows both the worksheet exported from WHOnet, this worksheet is generated by clicking on the “Save table” button and allows you to export both the data and the graphs shown by WHOnet itself, and the worksheet with transposed matrix that we created in order to import the data into R version 4.4.0 software.

Using MS Excel Version 2508 (build 19127.20192), the formula used to perform the data transposition within each table cell is the following:

 
  =IFERROR(
  INDEX('01.%RIS'!$C$9:$AU$90;
      MATCH        (1;
                       ('01.%RIS'!$F$9:$F$90=data_to_R!C$1)*
                       ('01.%RIS'!$J$9:$J$90=data_to_R!$A2)
                   ;0)
      ;15)
  ;"")
 

The formula was built with the following structure in mind:

1. Target Area –> INDEX('01.%RIS'!$C$9:$AU$90;; ... ; 15)
  - Identification of data source: from range C9:AU90 in the sheet '01.%RIS';
  - The row found from the MATCH(...) formula;
  - The column found from the 15^th^ column of that range (the column contains resistance data matched for organism, molecules and quarter).
2. Row Lookup –> MATCH(1; (...)*(...); 0)
  - MATCH(1; ...) looks for the first 1 (TRUE) in an array of 1s and 0s created by the following multiplication;
  - (...)*(...) multiplication defines the conditions to satisfy in order to make the relationship TRUE and therefore allow the data to be transposed into that cell where the formula was written;
  - (...) the brackets of point b contain the description of the criteria, in our formula they are:
    - '01.%RIS'!$F$9:$F$90=data_to_R!C$1 this equation searches among all antibiotics in the list F9:F90, the antibiotic in cell C1;
    - '01.%RIS'!$J$9:$J$90=data_to_R!$A2 this equation searches among all quarters in the list J9:J90, the quarter in cell A2;
  - If both conditions are satisfied, the row is correctly identified for the INDEX (...) function. If both conditions are not satisfied, an error message is returned.
3. INDEX(...; ...; 15)
  - Once the matching by row and by column is found, the formula extracts the value;
4. IFERROR(..., "")
  - If no match is found (i.e., the combination does not exist), the formula returns an empty string ("") instead of an error.

Sheet and cell references may need to be tailored to apply the formula to different exports from WHONET. Reference sheets can also be customized because the data exported by a researcher may vary based on sample size, time period, number of antimicrobials tested, and other variables the researcher would like to explore. The following table summarizes all sheet references mentioned above:

**Table A1:** Table of reference and descriptor for data transposition presented in Figure A1.

Reference	Descriptor
‘**01.%RIS**’	This Excel spreadsheet is generated by WHONET during the results export process described in step 3.5. By selecting the “Save table” option shown in Figure 2, the software creates a .csv file on the researcher’s computer containing the displayed information. (Figure A1, left side)
**data_to_R**	This sheet must be created by the researcher, preferably in the same file as sheet ‘**01.%RIS**’. Column A contains the x-axis labels of the graphs (quarters), while column B contains the quartile count, useful for regression analysis (1, 2, 3, 4). Row 1, for columns C and later, contains the names of the antimicrobials being studied (in this protocol, only levofloxacin was selected for demonstration purposes). (Figure A1, right side). The structure of this sheet is presented in Table 1**.**
**$C$9:$AU$90**	The range refers to all data exported from WHONET in .csv format. In this protocol, it includes the time variable (quarters, located in column J of sheet ‘**01.%RIS**’) and the antimicrobial variable (antibiotic names, located in column F of sheet ‘**01.%RIS**’).
**$F$9:$F$90**	The range refers to the column containing data on the tested antibiotics in the **‘01.%RIS’** sheet. These values are matched with the headers in the data_to_R sheet using the formula described in Appendix A, which extracts and translates the corresponding data for each antibiotic and quarter.
**$J$9:$J$90**	The range refers to the column containing data on specific quarter in the ‘**01.%RIS**’ sheet. These values are matched with the headers in the **data_to_R** sheet using the formula described in Appendix A, which extracts and translates the corresponding data for each antibiotic and quarter.
**C$1**	This part of the formula locks the selection to row 1 of the **data_to_R** sheet for the formula described above, enabling the formula to be dragged across the columns of the **data_to_R** sheet where the antimicrobial names are listed in the headers.
**$A2**	This part of the formula locks the selection to column A of the **data_to_R** sheet for the formula described above, enabling the formula to be dragged across the rows of the **data_to_R** sheet where quarters are listed in the labels.

## Appendix B

Below is provided the R code that can be used for the analysis (R version 4.4.0 and R-Studio 2025.05.0 Build 496). The highlighted strings in yellow must be customized with the paths of the files to be analyzed, and the folder where to save the output graphs. Both the path of the data file and the name of the sheet containing the data must be reported in quotation marks. Below is a facsimile example:

- “…path of your data file as organized in Appendix A…” ->
  ○ “C:/Users/User_Name/Desktop/Folder_Name/file.xlsx”
- “the name of the sheet which contains the data to import in R” ->
  ○ “data_to_import_into_R”

The R packages required for applying and running the script are as follows:

- readxl,
- ggplot2,
- broom,
- scales.

R script:

library(readxl) # Loads functions to read Excel (.xls and .xlsx) files into R.
library(ggplot2) # Loads tools to create advanced and customizable data visualizations using the grammar of graphics.
library(broom) # Provides functions to convert statistical analysis objects into tidy data frames for easy analysis and visualization.
library(scales) # Ensure 'scales' is loaded for rescale
 
# Read data
data <- read_excel("…path of your data file as organized in Appendix A…", sheet = "the name of the sheet which contains the data to import in R")
 
x_labels <- data[[1]]
time <- data[[2]] # This 'time' variable contains all original values from data[[2]]
colnames(data)[2] <- "Time"
 
# --- Plot Export Settings ---
# Define the output folder. It's good practice to create it if it doesn't exist.
# We will use a subfolder "Regression_Plots" within your Excel file's directory.
# Make sure the base path for your Excel file is correct for your system.
# Extract the directory of the Excel file
excel_dir <- dirname("…path of your data file as organized in Appendix A…")
output_dir <- file.path(excel_dir, "Regression_Plots") # The R output data will be placed in a folder called "Regression_Plots" in the same path as your datasheet.
 
# Create the folder if it doesn't exist. 'recursive = TRUE' creates parent folders if needed.
if (!dir.exists(output_dir)) {
  dir.create(output_dir, recursive = TRUE)
  message(paste("Output folder created:", output_dir))
    } else {
  message(paste("Output folder already exists:", output_dir))
    }
 
    # Start the loop to analyze each variable
    for (i in 3:ncol(data)) {
  variable_name <- colnames(data)[i]
  variable_values <- data[[i]]
 
  # Remove NAs for the model (this dataframe will be used ONLY for FITTING the model)
  df_model <- data.frame(time = time, y = variable_values)
  df_model <- na.omit(df_model)
 
  # Regression checks
  if (nrow(df_model) < 2 || sd(df_model$y) == 0) {
    message(paste("Variable skipped:", variable_name, "- insufficient data for regression"))
    next
  }
 
  # Linear model
  model <- lm(y ~ time, data = df_model)
  coeff <- coef(model)
  summary_model <- summary(model)
  r2 <- summary_model$r.squared
 
  if (nrow(coef(summary_model)) >= 2) {
    p_value <- coef(summary_model)[2, 4]
    formula_text <- paste0(
  "y = ", round(coeff[2], 2), "x + ", round(coeff[1], 2),
  "\nR\u00b2 = ", round(r2, 3), # Used \u00b2 for the squared symbol
  "\np = ", format.pval(p_value, digits = 3, eps = 0.001)
    )
  } else {
    formula_text <- "Invalid Model"
  }
 
  # Dataframe for points (uses the full original 'time' variable)
  df_plot <- data.frame(
    Time = time, # 'time' here is the global variable, includes all original values
    Label = factor(x_labels, levels = unique(x_labels)),
    Value = variable_values
  )
   
  # **NEW: Creating a dataframe for prediction over the entire 'time' range**
  # Use all unique and sorted 'time' values from the original dataset
  pred_time_values <- sort(unique(time))
  pred_df <- data.frame(time = pred_time_values)
   
  # Perform prediction of values and confidence interval
  predictions <- predict(model, newdata = pred_df, interval = "confidence")
  pred_data <- cbind(pred_df, as.data.frame(predictions))
  colnames(pred_data) <- c("time", "fit", "lwr", "upr") # Rename columns for clarity
   
  # Plot
  p <- ggplot() +
    # **NEW: Add geom_ribbon for the extended confidence interval**
    geom_ribbon(
      data = pred_data, # Use the new 'pred_data' dataframe
      aes(x = time, ymin = lwr, ymax = upr),
      fill = "grey80",
      alpha = 0.5 # Makes the interval slightly transparent
    ) +
    # **NEW: Add geom_line for the extended regression line**
    geom_line(
      data = pred_data, # Use the new 'pred_data' dataframe
      aes(x = time, y = fit),
      color = "black",
      linetype = "dashed",
      linewidth = 0.8 # Sets the line thickness (use 'linewidth' for ggplot2 >= 3.4.0)
    ) +
	 
    # Colorized points on the X-axis which will now use 'Time' (numeric from df_plot)
    geom_point(
      data = df_plot,
      aes(x = Time, y = Value, fill = Value), # 'Time' is numeric
      shape = 21,
      color = "black",
      size = 3,
      stroke = 1,
      na.rm = TRUE
    ) +
	 
    # Ensure the X-axis is continuous but with desired discrete labels
    scale_x_continuous(
      breaks = unique(df_plot$Time), # Numeric positions for breaks
      labels = unique(df_plot$Label) # Discrete labels for breaks
    ) +
	 
    scale_y_continuous(
      limits = c(0, 100),
      labels = function(x) paste0(x, "%")
    ) +
	 
    scale_fill_gradientn(
      colours = c("green", "goldenrod1", "red"),
      values = scales::rescale(c(0, 50, 100)),
      limits = c(0, 100),
      name = "Value"
    ) +
	 
    ggtitle(paste("Regression:", variable_name)) +
    xlab("Time (Quarter)") +
    ylab("Resistance %") +
	 
    annotate(
      "text",
      x = min(pred_data$time, na.rm = TRUE), # Use the minimum 'time' value from prediction
      y = 100,
      label = formula_text,
      hjust = 0, vjust = 1, # Top-left alignment
      size = 4, color = "black"
    ) +
	 
    theme_minimal() +
    theme(
      axis.text.x = element_text(angle = 45, hjust = 1), # Rotate X-axis labels
      plot.title = element_text(hjust = 0.5) # Center the plot title
    )
	 
  print(p) # Display the plot on screen
   
  # --- Export the plot as an image (PNG) ---
  # Create a safe filename for the variable
  safe_variable_name <- gsub("[^A-Za-z0-9_\\-]", "_", variable_name) # Removes invalid characters
  filename <- paste0(safe_variable_name, "_regression_plot.png")
  filepath <- file.path(output_dir, filename)
   
  ggsave(
    filename = filepath,
    plot = p,
    width = 10,  # Plot width in inches
    height = 6,  # Plot height in inches
    dpi = 300    # Resolution in DPI (Dots Per Inch) - 300 is standard for high quality
  )
  message(paste("Plot saved:", filepath))
}
